# En Bloc Sutureless Patch Repair of the Inferior Vena Cava and Right Hepatic Vein in a Patient with Invasive Renal Cell Carcinoma: A Case Report

**DOI:** 10.70352/scrj.cr.26-0531

**Published:** 2026-11-10

**Authors:** Keita Amakura, Shin Yajima, Daisuke Yoshioka, Atsunari Kawashima, Shinichiro Hasegawa, Takuji Kawamura, Ai Kawamura, Yusuke Misumi, Shigeru Miyagawa

**Affiliations:** 1 Department of Cardiovascular Surgery, The University of Osaka, Suita, Osaka, Japan; 2 Department of Urology, The University of Osaka, Suita, Osaka, Japan; 3 Department of Gastroenterological Surgery, The University of Osaka, Suita, Osaka, Japan

**Keywords:** sutureless patch repair, autologous pericardium, renal cell carcinoma

## Abstract

**INTRODUCTION:**

In patients with invasive renal cell carcinoma, extensive involvement of the inferior vena cava (IVC) and right hepatic vein presents a unique surgical challenge. We report a case of a man with invasive renal cell carcinoma of the left kidney who developed massive hemorrhage and hypovolemic shock due to right hepatic vein laceration during surgery, which necessitated an emergency repair procedure. This procedure was effective and might be useful in other patients with invasive renal cell carcinoma with IVC and hepatic vein involvement.

**CASE PRESENTATION:**

A 72-year-old man presented with severe acute left-sided epigastric pain. Plain abdominal CT and MRI revealed an enlarged left kidney with a low-density mass extending from the left renal vein to the IVC. Renal biopsy revealed renal cell carcinoma of the left kidney. The patient was treated with neoadjuvant therapy and underwent surgery. A median laparotomy was performed to expose and dissect the IVC. During the Kocher maneuver to mobilize the liver, the right hepatic vein was lacerated because the venous wall had been weakened by tumor thrombus. This led to massive hemorrhage and hypovolemic shock, necessitating an emergency repair procedure. The IVC defect and transected right hepatic vein were reconstructed en bloc using an autologous pericardial patch. The upper half of the patch was sutured directly to the liver parenchyma (not to the right hepatic vein), whereas the lower half was attached to the residual IVC wall. After vascular reconstruction, the left kidney and associated renal veins were excised. Postoperative contrast-enhanced abdominal CT revealed patent blood flow from the right hepatic vein through the patch to the IVC. Pre-existing small pulmonary nodules subsequently increased in size and were considered likely metastatic lesions. The patient died of respiratory failure on POD 120.

**CONCLUSIONS:**

Sutureless patch repair offers an alternative to conventional grafting methods and may be useful in cases of invasive carcinoma that require combined reconstruction of the hepatic veins and IVC.

## Abbreviation

IVC
: inferior vena cava

## INTRODUCTION

Renal cell carcinoma can occasionally invade the IVC necessitating combined resection and reconstruction. In cases where the tumor extends extensively, cardiopulmonary bypass (CPB) support is sometimes required during surgery to maintain hemodynamic stability. Several techniques are reported for IVC reconstruction, including replacement with a Dacron graft^1,2)^ or repair using bovine^3,4)^ or autologous pericardium.^5)^ However, extensive involvement of the IVC and right hepatic vein poses a unique surgical challenge.

Here, we describe an en bloc sutureless patch repair of the IVC and right hepatic vein in a patient with invasive renal cell carcinoma. This approach offers an alternative to conventional grafting methods.

## CASE PRESENTATION

A 72-year-old man presented with anorexia and severe acute onset left-sided epigastric pain. Plain abdominal CT revealed a swollen left kidney with a low-density mass extending from the left renal vein to the IVC, reaching the level of the diaphragm. Subsequent abdominal MRI confirmed these findings.

Renal biopsy revealed renal cell carcinoma with necrosis. Based on these findings, the patient was diagnosed with cT3c renal cell carcinoma with IVC invasion and treated with pembrolizumab plus lenvatinib combination therapy for a total of 5 courses, administered every 3 weeks as neoadjuvant therapy. The extent of the tumor was reassessed preoperatively using CT, MRI, and bone scintigraphy. The imaging revealed a slight reduction in the tumor size (**Fig. 1A** and **1B**) and showed that the tumor thrombus extended from the left renal vein into the IVC up to the level of the diaphragm. The distal IVC was already completely occluded by the tumor thrombus preoperatively. Small pulmonary nodules were also present before surgery, but they could not be definitively diagnosed as metastatic lesions; therefore, no definite metastatic disease was identified. Given the absence of definite metastatic disease, surgery was planned to achieve complete resection. The treatment plan was discussed in a multidisciplinary conference involving urology, gastrointestinal surgery, and cardiovascular surgery specialists, and the neoadjuvant therapy and surgical strategy were determined collaboratively.

**Fig. 1:**
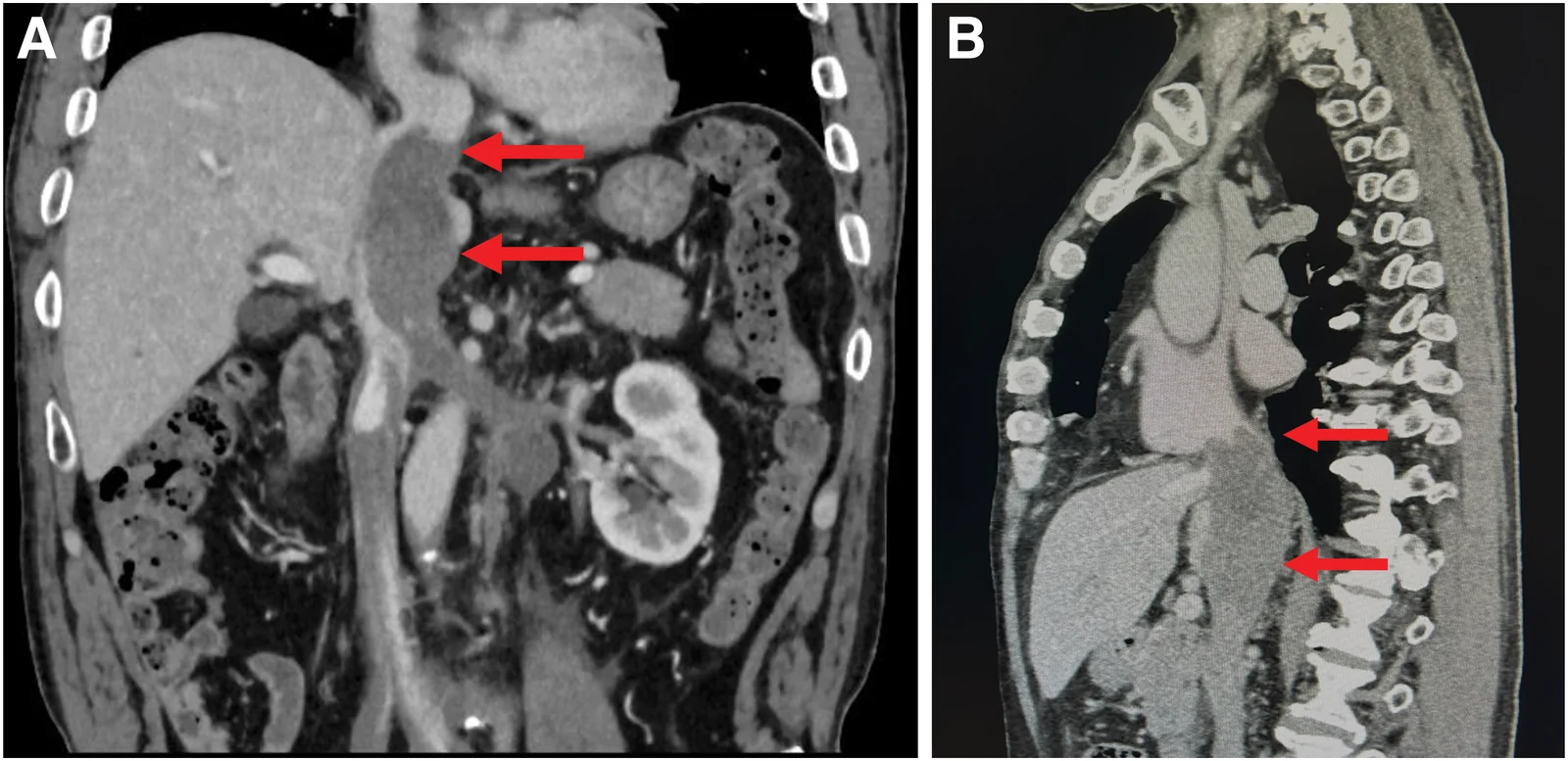
(**A**) and (**B**) Abdominal contrast-enhanced CT image taken after neoadjuvant chemotherapy showing a tumor of the left kidney extending from the left renal vein into the IVC, up to the level of the diaphragm (red arrows). IVC, inferior vena cava

The initial surgical plan was tumor resection through an abdominal approach without extracorporeal circulation, with escalation to intrapericardial IVC control and CPB if adequate vascular control could not be obtained. To permit rapid initiation of CPB if required, the right internal jugular vein and the right femoral artery were cannulated prophylactically before laparotomy. A staged strategy to minimize the duration of hepatic inflow interruption was also planned.

A median laparotomy was performed to expose and dissect the IVC. The left renal vein and the hepatic hilar IVC were secured with vascular control tape. Intraoperative findings, including direct ultrasonographic assessment, showed that the tumor was firmly adherent to the IVC wall and could not be sufficiently mobilized. During the Kocher maneuver to mobilize the liver, the right hepatic vein was inadvertently lacerated because the fragile venous wall was weakened by the pre-existing tumor thrombus obstructing the venous lumen. This resulted in massive, uncontrollable hemorrhage (**Fig. 2A**) and consequent hypovolemic shock. Hemostasis was attempted by clamping the IVC above the diaphragm via a lower sternotomy and at the lower hepatic hilar level, together with total hepatic pedicle occlusion at the hepatic hilum. However, bleeding remained uncontrolled. Using the prophylactically placed cannulas, CPB was promptly established with venous drainage from the right internal jugular vein and lower IVC and arterial return via the right femoral artery.

**Fig. 2:**
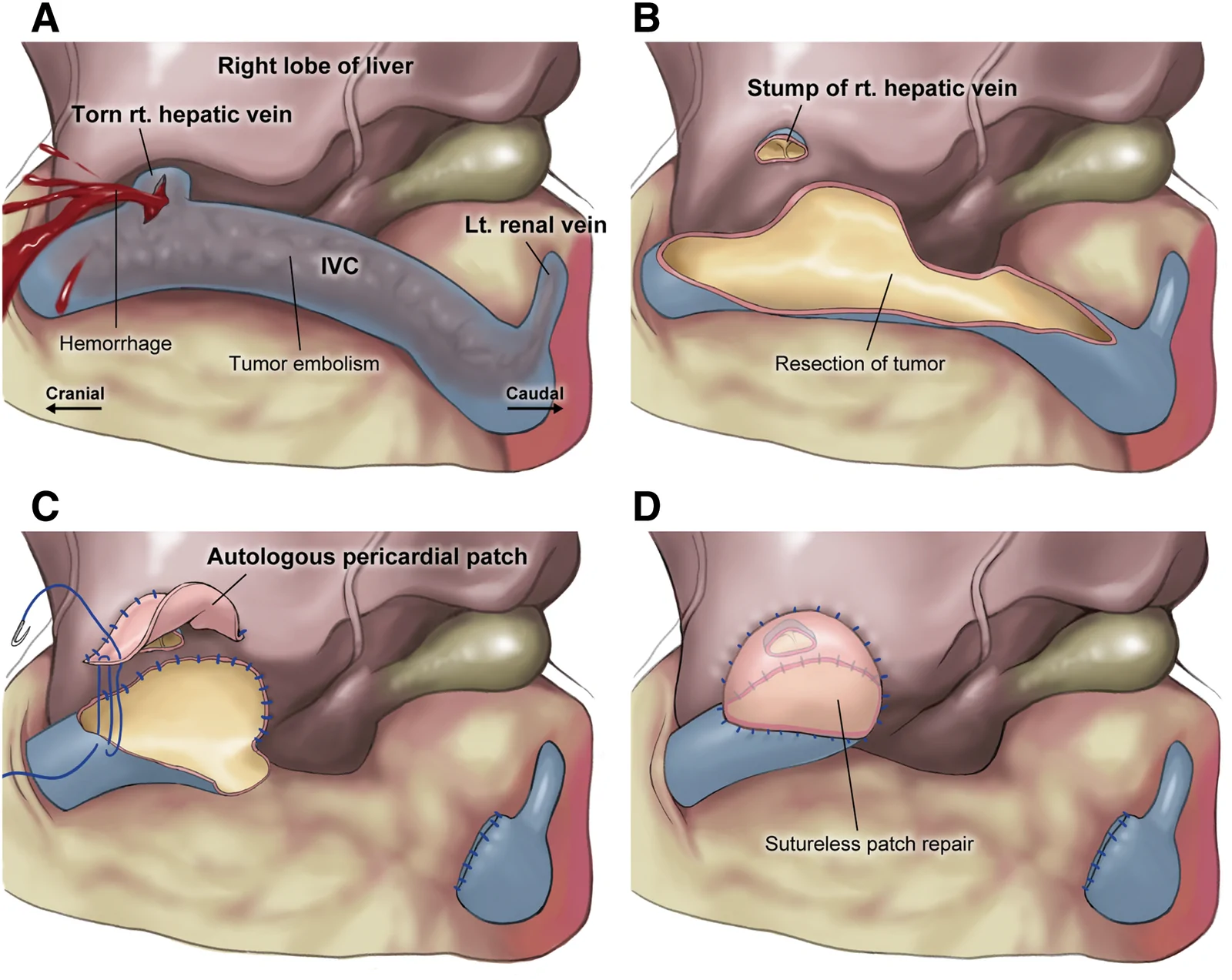
Operative scheme. (**A**) A laceration of the right hepatic vein, caused by a fragile venous wall weakened by tumor embolization, resulting in massive, uncontrollable hemorrhage. (**B**) Extensive resection of the IVC wall and transected right hepatic vein owing to its firm adhesion to the tumor. (**C**) En-bloc reconstruction of the IVC defect and right hepatic vein using an autologous pericardial patch. The patch’s upper half was sutured directly to the liver parenchyma, while the lower half was anchored to the residual IVC wall. (**D**) Final appearance of the sutureless patch repair. IVC, inferior vena cava

The laceration progressively enlarged and was nearly detached from the origin of the right hepatic vein. Thus, reconstruction in conjunction with IVC repair, rather than individual repair, was required. To achieve complete tumor removal, the IVC wall was resected extensively because of firm adhesion between the tumor and the vessel (**Fig. 2B**), and the distal IVC was closed as a blind end. Additional thorough dissection around the IVC and diaphragm was performed to free the IVC from surrounding structures and reposition it closer to the liver. The resulting IVC defect and the right hepatic vein were reconstructed en bloc using an autologous pericardial patch. The upper half of the patch was sutured directly to the liver parenchyma rather than to the right hepatic vein, whereas the lower half was sutured to the residual IVC wall (**Fig. 2C** and **2D**). After vascular reconstruction, the left kidney and associated renal veins were excised (**Fig. 3**).

**Fig. 3:**
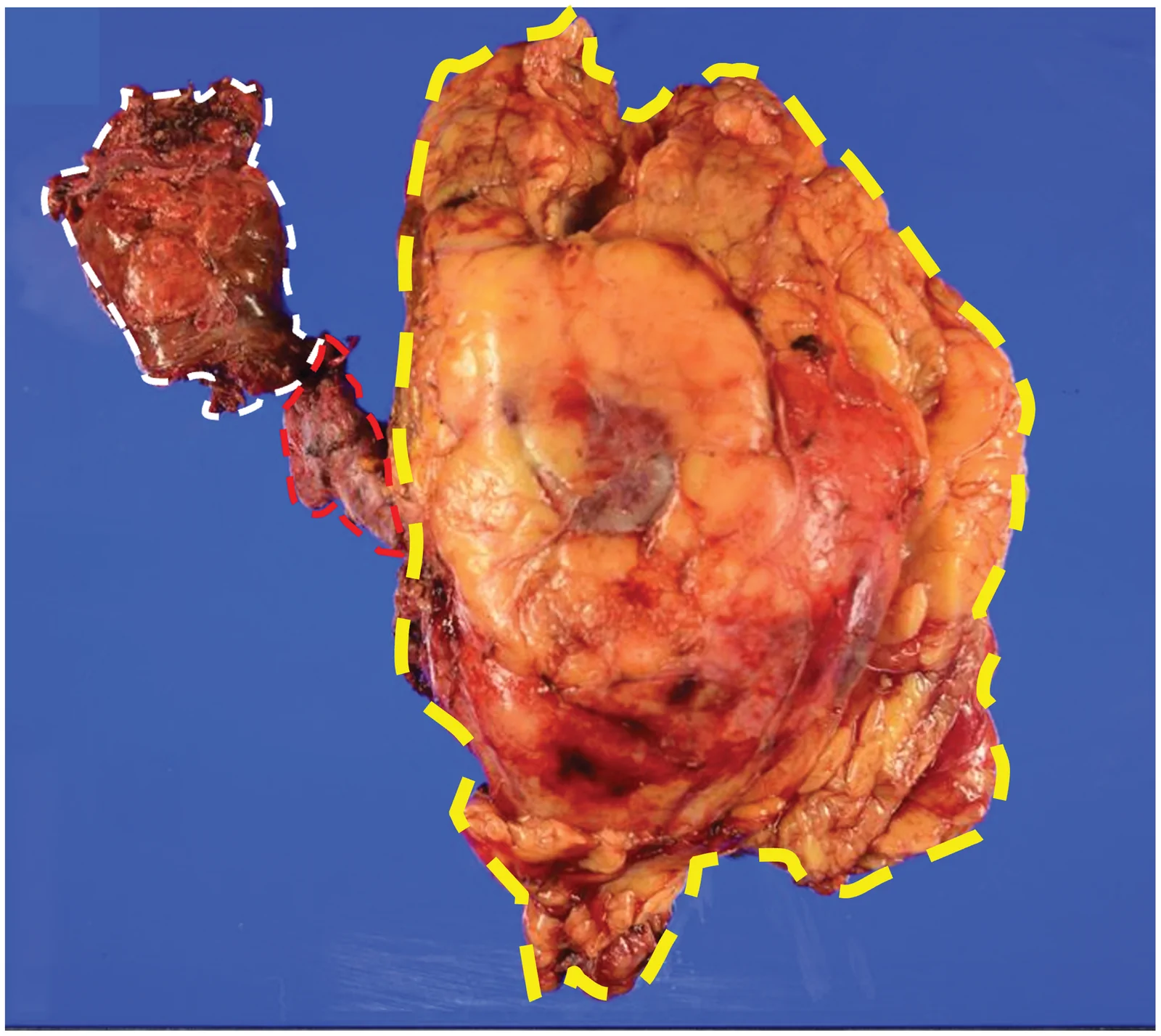
Macroscopic pathological image of the left kidney (yellow dashed line) showing continuity with tumor embolization extending into the left renal vein (red dashed line) and the IVC (white dashed line). IVC, inferior vena cava

Postoperative contrast-enhanced abdominal CT revealed patent blood flow from the right hepatic vein through the patch to the IVC (**Fig. 4**), although mild narrowing of the reconstructed IVC was observed. Hepatic blood flow was regularly assessed using ultrasonography, and no apparent abnormalities in hepatic perfusion or venous drainage were detected. The liver enzyme levels returned to near-normal values by POD 14 (**Fig. 5**). The patient required temporary postoperative dialysis, which was subsequently discontinued following renal function recovery. Lower-body edema was initially observed after surgery but gradually resolved. The small pulmonary nodules noted preoperatively increased in size during the postoperative period and were considered metastatic lesions. The patient died of respiratory failure on POD 120.

**Fig. 4:**
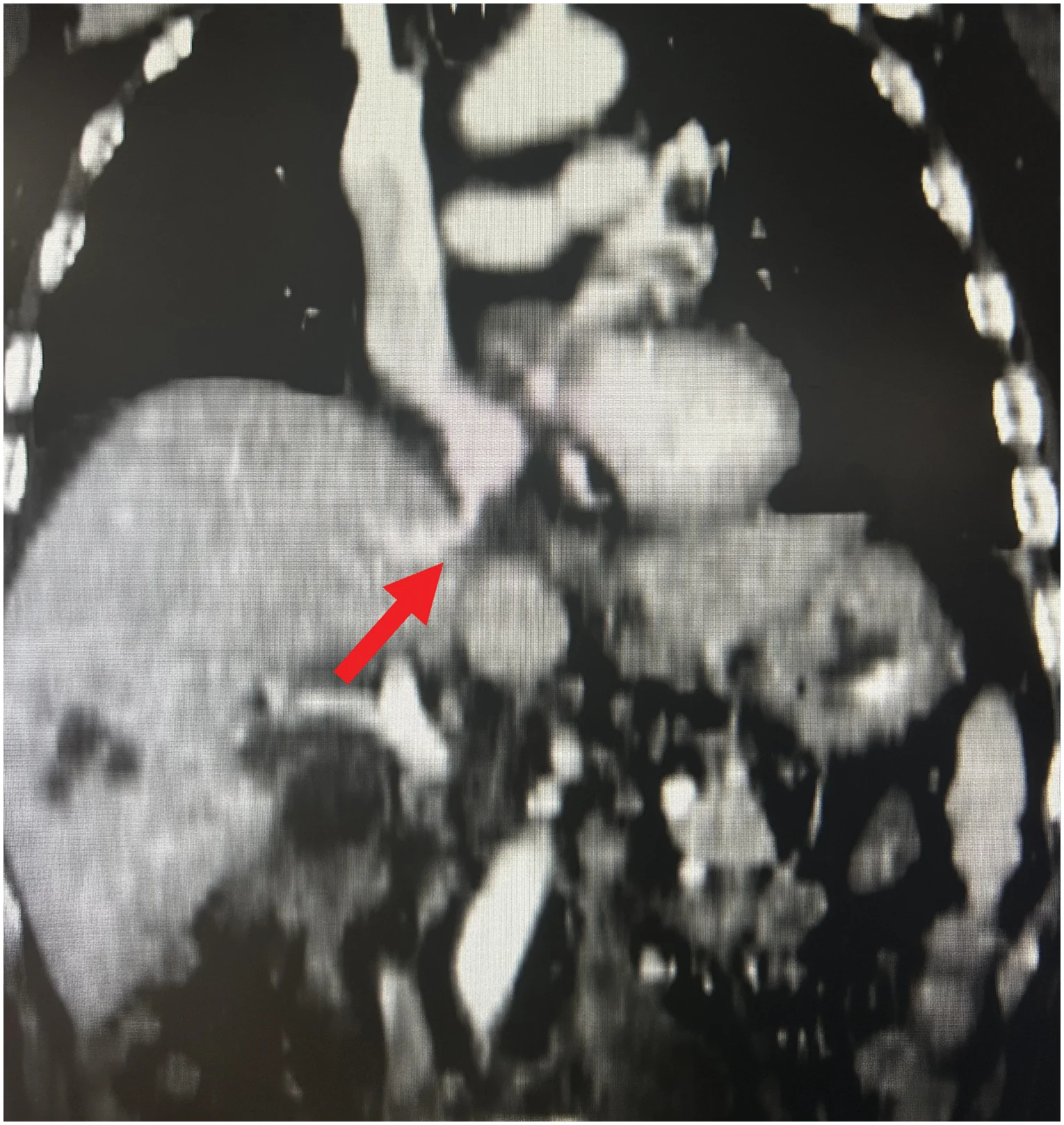
Postoperative contrast-enhanced abdominal CT images in coronal view showing patent blood flow from the right hepatic vein, through the patch, into the IVC. The red arrow shows a patent right hepatic vein. IVC, inferior vena cava

**Fig. 5:**
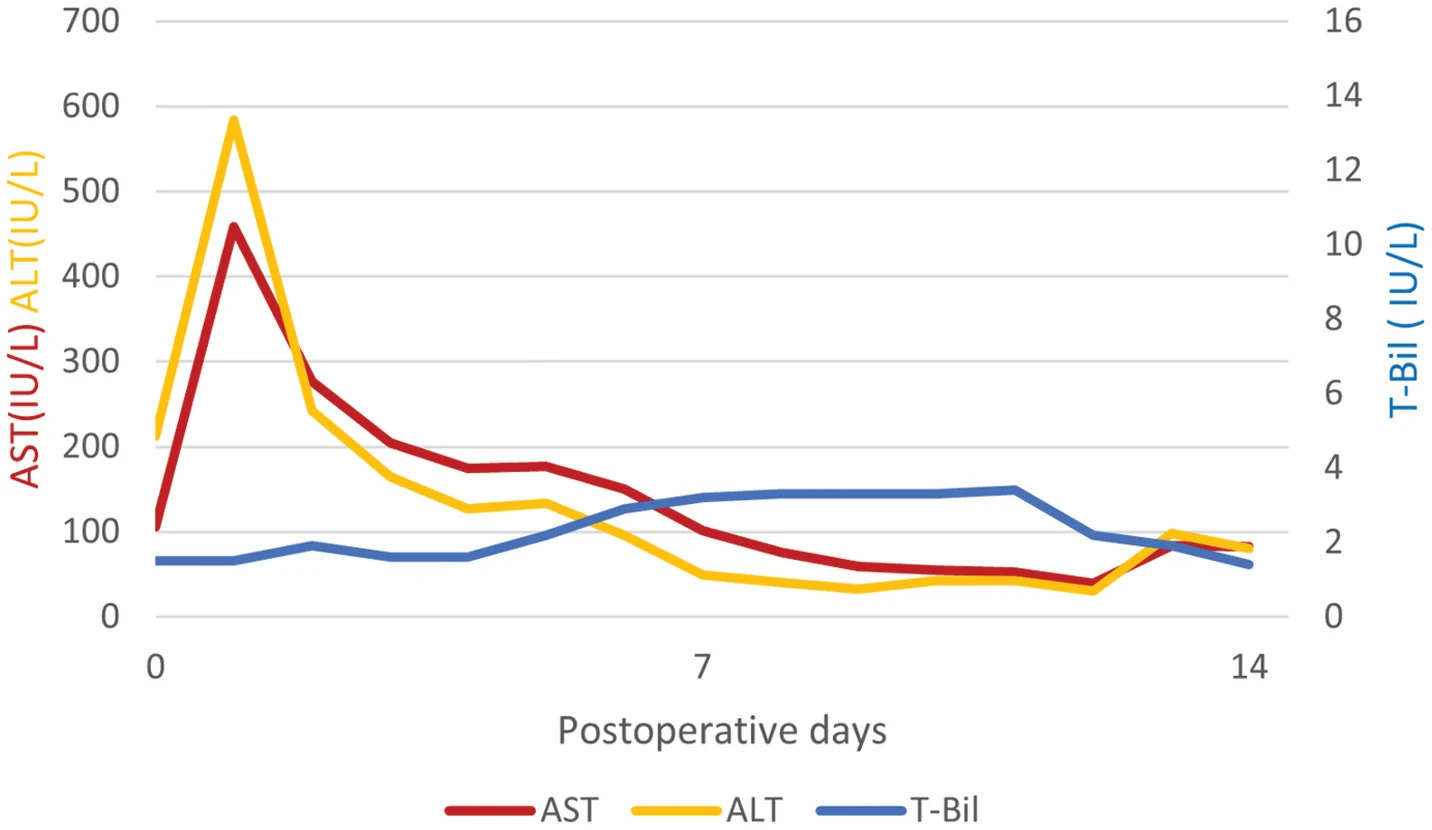
Postoperative changes in liver enzymes. AST (red line), ALT (yellow line), and total bilirubin (blue line) levels returned to nearly normal by POD 14. ALT, alanine aminotransferase; AST, aspartate aminotransferase; T-bil, total bilirubin

## DISCUSSION

The novelty of the present case report lies in the unique en bloc reconstruction of both the IVC and the right hepatic vein. Reconstruction of the right hepatic vein was crucial for preventing hepatic congestion, which would have otherwise necessitated a right lobectomy. Some surgeons may consider individual repair of the IVC and right hepatic vein using direct vascular anastomosis or an interposed vein or Dacron graft. However, such reconstructions may be susceptible to stenosis, kinking, or occlusion after the liver is repositioned to its anatomical location.

Sutureless patch repair was used in congenital heart surgery^6,7)^ and pulmonary vein reconstruction.^8)^ In this technique, sutures are placed in the surrounding tissue rather than directly in the target vessel to reduce the risk of vessel stenosis or kinking. In the present reconstruction, the hepatic venous component followed this sutureless concept because the patch was anchored to the liver parenchyma than directly to the right hepatic vein. This approach was selected to provide a broad and flexible hepatic venous outflow pathway. The mild narrowing seen on postoperative CT appeared to involve the reconstructed IVC rather than the right hepatic vein and may have resulted from extrinsic compression by the weight and anatomical position of the liver after repositioning. The absence of abnormalities in hepatic perfusion or venous drainage on serial ultrasonography supports preserved functional outflow despite this finding. By contrast, direct vascular anastomosis of the right hepatic vein could have been more susceptible to kinking or distortion after repositioning of the liver, potentially causing severe hepatic venous outflow obstruction. Thus, the sutureless patch may have helped reduce the risk of complete venous outflow obstruction. Although anchoring the patch to the liver parenchyma raised concerns about liver injury or bile leakage, postoperative liver enzymes and total bilirubin decreased to near-normal levels within 2 weeks.

Although reconstruction of the entire IVC using the patch might have been technically possible, the distal IVC was completely occluded by the tumor thrombus preoperatively. Accordingly, restoration of direct IVC continuity was not considered essential, and closure of the distal end as a blind end was considered an acceptable option in this emergent setting. The subsequent clinical course also did not suggest persistent clinically significant impairment of lower-body venous drainage: postoperative dialysis could be discontinued after renal recovery, and lower-body edema gradually improved. We, therefore, considered the transient renal dysfunction to be more consistent with the substantial intraoperative blood loss and hemodynamic instability than with closure of the distal IVC.

Although the tumor thrombus reached the level of the diaphragm, sternotomy was not performed at the outset because, unless reconstruction of the right atrium itself is required, vascular control and IVC reconstruction can generally be approached from the abdomen. We anticipated that the tumor thrombus might be removed through an IVC incision below the diaphragm if the tumor could be mobilized and the IVC controlled above the tumor. If more proximal control became necessary, partial division of the diaphragm could extend the operative field into the thoracic cavity and provide access to the intrapericardial IVC, with partial sternotomy added if required. Thus, our planned escalation strategy was to use intrapericardial IVC control and CPB only when adequate vascular control could not be achieved through the abdominal approach. Prophylactic cannulation before laparotomy was performed to allow this escalation to CPB without delay in the event of circulatory collapse.

To minimize hepatic congestion and ischemic injury, our strategy was to limit hepatic inflow occlusion to within approximately 15 minutes whenever possible. The tumor thrombus cranial to the hepatic veins had to be removed first, followed by reconstruction or closure of the IVC down to the level immediately below the hepatic vein confluence during temporary hepatic inflow interruption. Hepatic blood flow would then be restored before removal of the remaining tumor thrombus caudal to the hepatic veins. This staged approach was intended to avoid prolonged hepatic ischemia and congestion during tumor resection and vascular reconstruction.

There were pre-existing small pulmonary nodules that could not be definitively diagnosed as metastatic lesions and these subsequently increased in size during the postoperative period. We, therefore, considered the postoperative pulmonary metastases to most likely represent progression of pre-existing pulmonary lesions that were likely metastatic than metastases newly induced or directly caused by the surgical procedure. Although we cannot completely exclude the possibility that the physiological stress associated with major surgery or CPB may have influenced tumor progression, there was no clinical evidence suggesting a direct causal relationship between the surgical procedure itself and the development of pulmonary metastases in this patient.

Based on our experience, we recommend that such extensive reconstructive procedures be performed only in high-volume centers with strong multidisciplinary collaboration. Careful patient selection, including comprehensive systemic disease assessment and evaluation of tumor response to neoadjuvant therapy, should be performed before undertaking aggressive surgical resection. Our “sutureless patch technique” may be applicable to cases requiring extensive reconstruction of the IVC and adjacent major vessels while maintaining vascular continuity.

## CONCLUSIONS

Sutureless patch repair may be useful in cases of invasive abdominal cancer that require combined reconstruction of the hepatic vein and IVC. This method offers a feasible alternative to conventional grafting methods.

## DECLARATIONS

### Availability of data and materials

All data supporting the findings of this study are available in the article.
